# Coexistence of Multidrug Resistance and Virulence in a Single Conjugative Plasmid from a Hypervirulent Klebsiella pneumoniae Isolate of Sequence Type 25

**DOI:** 10.1128/msphere.00477-22

**Published:** 2022-12-06

**Authors:** Peiwen Xia, Miao Yi, Yaling Yuan, Jinzhu Huang, Bingxue Yang, Jiajia Liao, Zijun Dang, Shengli Luo, Yun Xia

**Affiliations:** a Department of Laboratory Medicine, The First Affiliated Hospital of Chongqing Medical University, Chongqing, China; JMI Laboratories

**Keywords:** conjugative, cotransmission, *bla*_KPC-2_, *iuc* operon, *Klebsiella pneumoniae*, ST25

## Abstract

Carbapenem-resistant hypervirulent Klebsiella pneumoniae (CR-hvKP) has received considerable attention. Typically, the genetic elements that confer virulence are harbored by nonconjugative plasmids. In this study, we report a CR-hvKP strain, CY814036, of high-risk sequence type 25 (ST25) and the K2 serotype, which is uncommon among K. pneumoniae isolates but caused serious lung infection in a tertiary teaching hospital in China. Whole-genome sequencing (WGS) revealed a rare conjugative plasmid, pCY814036-iucA, carrying a virulence-associated *iuc* operon (*iucABCD-iutA*) coding for aerobactin and determinants of multidrug resistance (MDR), coexisting with a conjugative *bla*_KPC-2_-bearing plasmid, pCY814036-KPC2, in the same strain. A conjugation assay showed that pCY814036-iucA and pCY814036-KPC2 could be efficiently cotransmitted from CY814036 to Escherichia coli EC600. Further phenotypic investigation, including antimicrobial susceptibility tests, serum resistance assays, and mouse infection models, confirmed that pCY814036-iucA was capable of cotransferring multidrug resistance and hypervirulence features to the recipient. pCY814036-KPC2 also conferred resistance to antibiotics, including β-lactams and aminoglycosides. Overall, the rare coexistence of a conjugative MDR-virulence plasmid and a *bla*_KPC-2_-bearing plasmid in a K. pneumoniae isolate offers a possible mechanism for the formation of CR-hvKP strains and the potential to significantly accelerate the propagation of high-risk phenotypes.

**IMPORTANCE** The increased reporting of carbapenem-resistant hypervirulent K. pneumoniae is considered a worrisome concern to human health care and has restricted the choice of effective antibiotics for clinical treatment. Moreover, virulence plasmids with complete conjugation modules have been identified, which evolved via homologous recombination. Here, we characterize an ST25 CR-hvKP strain, CY814036, harboring both a conjugative MDR-virulence plasmid and a *bla*_KPC-2_-bearing plasmid in China. This study highlights that the cotransmission of drug resistance and virulence plasmids increases therapeutic difficulties and worsens clinical prognoses. Also, active surveillance of the conjugative MDR-virulence plasmid is necessary.

## INTRODUCTION

Historically, the two pathotypes of Klebsiella pneumoniae responsible for hypervirulence (hypervirulent K. pneumoniae [hvKP]) and carbapenem resistance (carbapenem-resistant K. pneumoniae [CRKP]), respectively, have been regarded as belonging to nonoverlapping populations. Over the past few years, however, the rapid emergence of carbapenem-resistant hypervirulent K. pneumoniae (CR-hvKP) has received increasing attention as a severe challenge to human health (1). Unlike CRKP and hvKP, CR-hvKP not only can cause severe invasive infections but also has extremely limited the options for antimicrobial therapy (2, 3). Geographically, CR-hvKP has been reported across several continents, including Europe, Africa, and South and North America, but has been found mostly in Asia (4). In 2016, there was a deadly ventilator-associated pneumonia epidemic brought on by sequence type 11 (ST11) CR-hvKP in a Chinese hospital, signifying serious challenges for human health and infection control (2). Notably, a multicenter analysis in 2019 indicated that ST11 CR-hvKP was most frequently reported from China (5). The emergence of CR-hvKP of other STs (e.g., ST23, ST65, and ST15) has also been described (6–8).

The global dissemination of mobile genetic elements (MGEs), including plasmids, insertion sequences (ISs), and transposons (Tns), causes the horizontal gene transfer (HGT) of resistance and virulence phenotypes and, thus, is mediating the evolution of Klebsiella pneumoniae strains (9, 10). CR-hvKP is commonly considered to have evolved in two directions: (i) a CRKP strain acquiring virulence factors and (ii) an hvKP strain developing carbapenem resistance phenotypes. There are many data available showing that most acquired antimicrobial resistance (AMR) genes are associated with self-transmissible plasmids (11), whereas the traditional pK2044-like virulence plasmids of hvKP have been seen as nonconjugative, without complete conjugative modules, and also carry no antimicrobial resistance determinants. Subsequently, Xu et al. confirmed the mobilization of these plasmids to access new hosts via self-transferable IncF plasmids in the same host cell (12). Recently, however, several conjugative virulence plasmids have been shown to feature genes encoding aerobactin (*iucABCD-iutA*) and/or regulators of mucoid phenotype A (plasmid-borne *rmpA* [p-*rmpA*] and p-*rmpA2*) (13–15). Additionally, Li et al. observed an IncHI1B plasmid, p17-15-vir, in an ST15 K. pneumoniae strain, which was characterized as a conjugative multidrug resistance (MDR)-virulence plasmid, whereas such plasmids are still uncommon (8).

In this study, we characterized an ST25/K2 serotype CR-hvKP strain isolated from a clinical patient, which harbored a conjugative MDR-virulence plasmid and a *bla*_KPC-2_-bearing resistance plasmid. We used whole-genome sequencing (WGS) to reveal the genetic basis of the strain, and corresponding experiments were then employed to explore the transferability of the plasmids and confirm the relevant phenotype.

## RESULTS

### Clinical and phenotypic characteristics of K. pneumoniae strain CY814036.

K. pneumoniae strain CY814036 was isolated from a sputum sample of a 91-year-old female patient with diabetes and chronic obstructive pulmonary disease (COPD) who was hospitalized in the intensive care unit (ICU) of the First Affiliated Hospital of Chongqing Medical University in 2018. The patient was diagnosed with cerebral infarction and subsequently transferred to the ICU due to progression of the disease. During the course of treatment, the patient developed fever and cough with sputum production, which was empirically treated with cefoxitin, but the patient’s symptoms did not improve significantly. Subsequently, etimicin was added for combination therapy based on the results of antibiotic susceptibility tests for the K. pneumoniae isolate CY814036, but this patient remained infected and eventually died of respiratory and circulatory failure due to massive cerebral infarction. Unfortunately, we did not observe any clue regarding the transmission of K. pneumoniae CY814036 according to the patient’s medical records. Antimicrobial susceptibility tests were performed, and K. pneumoniae CY814036 displayed resistance to all β-lactam, aminoglycoside, and fluoroquinolone antibiotics but remained susceptible to ceftazidime-avibactam and tigecycline (Table 1). Notably, we observed a viscous string of around 20 mm (>5 mm) generated by CY814036, which indicated that this strain possessed a hypermucoviscosity phenotype. PCR analysis showed that strain CY814036 carried both the resistance gene *bla*_KPC-2_ and the virulence genes *iucA*, *iroB*, and *peg-344*.

**TABLE 1:** Antimicrobial susceptibility profiles of hypervirulent K. pneumoniae CY814036 and its transconjugants

Strain identifier	Description	MIC (mg/L)^*a*^										
		CAZ	CRO	FEP	IPM	MEM	CAZ-AVI	TGC	ATM	CIP	LEV	AK
CY814036	ST25/K2 CR-hvKP	16	≥128	8	8	8	≤0.5/4	2	128	1	2	≥512
EC600	Rifampicin-resistant E. coli	≤0.5	≤0.125	≤0.06	0.125	≤0.06	≤0.5/4	0.25	≤0.25	0.125	0.25	4
EC600-TC_a	Transconjugant with pCY814036-iucA and pCY814036-KPC2	16	≥128	8	4	4	≤0.5/4	0.25	128	1	2	≥512
EC600-TC_b	Transconjugant with pCY814036-KPC2	32	≥128	8	4	4	≤0.5/4	0.25	128	0.125	0.25	≥512
ATCC 25922	Control strain	≤0.5	≤0.125	≤0.06	0.25	≤0.06	≤0.5/4	0.25	≤0.25	≤0.016	≤0.06	4

a CAZ, ceftazidime; CRO, ceftriaxone; FEP, cefepime; IPM, imipenem; MEM, meropenem; CAZ-AVI, ceftazidime-avibactam; TGC, tigecycline; ATM, aztreonam; CIP, ciprofloxacin; LEV, levofloxacin; AK, amikacin.

### Genetic characterization of CR-hvKP strain CY814036.

To understand the genetic basis of this CR-hvKP isolate, whole-genome sequencing (WGS) was applied using the Pacific Biosciences (PacBio) RS II single-molecule real-time (SMRT) and Illumina (San Diego, CA) HiSeq platforms. Our analysis showed that CY814036 belonged to sequence type 25 (*gapA-infB-mdh-pgi-phoE-rpoB-tonB*, allele number 2-1-1-1-10-4-13) and capsular serotype K2, which was not the major ST11 clone reported for carbapenemase-producing hvKP isolates in China (5). The whole CY814036 genome assembly consisted of a chromosome of 5,423,834 bp and two circular plasmids, pCY814036-iucA (GenBank accession no. CP093152) and pCY814036-KPC2 (GenBank accession no. CP093153), which is consistent with the results of S1 nuclease pulsed-field gel electrophoresis (S1-PFGE) (see Fig. 3b).

Using the Virulence Factors Database (VFDB) (http://www.mgc.ac.cn/VFs/main.htm), we found a large number of virulence-associated factors that were carried by strain CY814036, including genes encoding aerobactin (*iucABCD-iutA*), salmochelin (*iroBCDEN*), yersiniabactin (*fyuA*, *irp1*, *irp2*, and *ybtAEPQSTUX*), Ent siderophore (*entABCDEFS*), chromosomal *rmpA* (c-*rmpA*), and type 3 fimbriae (*mrkABCDFHIJ*), etc. Among them, the key hypervirulence factor aerobactin encoded by the *iucABCD-iutA* genes was identified in plasmid pCY814036-iucA (16), while the rest of the virulence genes were located on the chromosome. Although strain CY814036 was phenotypically hypermucoviscous, genes coding for regulators of the mucoid phenotype (p-*rmpADC* and/or p-*rmpA2*) were absent. In addition to the virulence factors, 25 antimicrobial resistance genes (19 plasmid borne and 5 chromosome mediated) were identified in CY814036 using the Comprehensive Antibiotic Resistance Database (CARD) (https://card.mcmaster.ca/analyze/rgi). Notably, most of the significant drug resistance genes were harbored by both pCY814036-iucA and pCY814036-KPC2, which mediated resistance to β-lactams, aminoglycosides, sulfonamide, fluoroquinolones, tigecycline, and rifampicin, etc. The resistance and virulence genes of CY814036 are shown in Table 2. Complete information on the virulence factors and related genes on the chromosome can also be found in Table S1 in the supplemental material.

**TABLE 2:** Molecular characteristics of hypervirulent K. pneumoniae CY814036 and its plasmids

Feature	Value for CY814036		
	Chromosome	pCY814036-iucA	pCY814036-KPC2
Size (bp)	5,423,834	257,343	140,105
Incompatibility group	—^*a*^	IncFIB_K_/IncFII_K_	IncFII/IncR
Resistance determinants	*bla*_SHV-11_, *oqxA*, *rsmA*, *emrR*, *adeF*, *baeR*, *marA*, *msbA*, *adeF*, *marA*	*bla*_OXA-10_, *tet*(A), *bla*_LAP-2_, *ANT(3″)-IIa*, *aph(3″)-Ib* (*strA*), *aph(6)-Id* (*strB*), *floR*, *cmlA5*, *qnrS1*, *arr-2*, *sul2*, *dfrA14*	*bla*_KPC-2_, *bla*_CTX-M-65_, *bla*_TEM-1_, *rmtB*
Virulence factor(s) (gene[s])^*b*^	Salmochelin (*iroBCDEN*), yersiniabactin (*fyuA*, *irp1*, *irp2*, and *ybtAEPQSTUX*), Ent siderophore (*entABCDEFS*), regulator of the mucoid phenotype (c-*rmpA*), type 3 fimbriae (*mrkABCDFHIJ*), type 1 fimbriae (*fimABCDEFGHIK*), RcsAB (*rcsAB*), PEG344 (*peg-344*), AcrAB (*acrAB*)	Aerobactin (*iucABCD-iutA*)	—
Conjugative modules	—	*oriT* region, relaxase gene, T4CP gene, gene cluster for the T4SS	*oriT* region, relaxase gene, gene cluster for the T4SS

a —, nonexistent information.

b Only major virulence factors and related genes on the chromosome are listed. Complete information can be found in Table S1 in the supplemental material.

10.1128/msphere.00477-22.2 TABLE S1 Virulence factors, related genes, and locations of genes on the chromosome of K. pneumoniae CY814036. Putative virulence factors were identified using the Virulence Factors Database (VFDB). Download Table S1, PDF file, 0.4 MB. Copyright © 2022 Xia et al. 2022 Xia et al. https://creativecommons.org/licenses/by/4.0/ This content is distributed under the terms of the Creative Commons Attribution 4.0 International license.

### Identification of conjugative MDR-virulence plasmid pCY814036-iucA.

**FIG 1:**
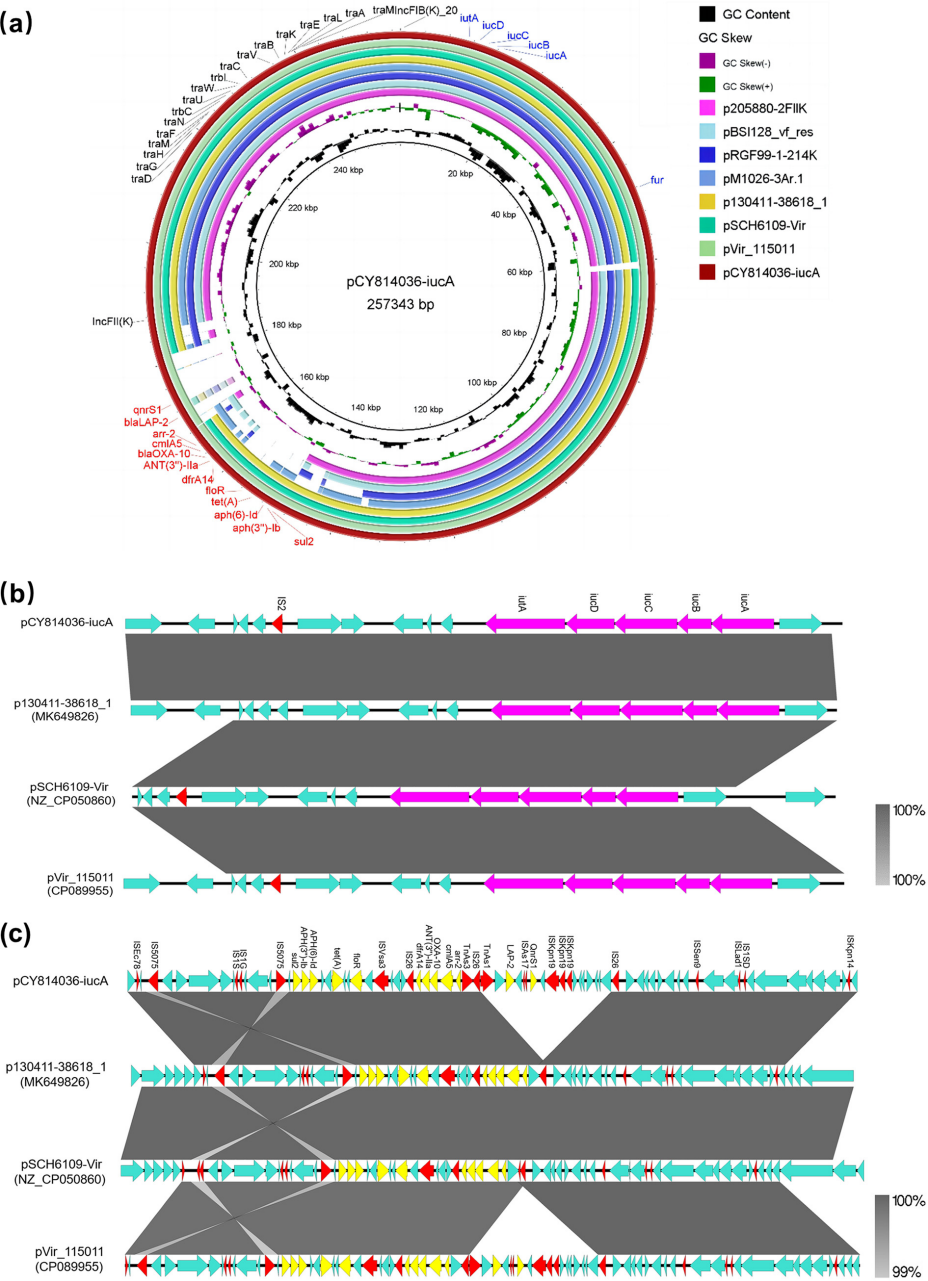
(a) Genomic alignment of pCY814036-iucA (GenBank accession no. CP093152) with other *iucA*-harboring virulence plasmids, including pVir_115011 (accession no. CP089955), pSCH6109-Vir (accession no. NZ_CP050860), p130411-38618_1 (accession no. MK649826), pM1026-3Ar.1 (accession no. NZ_CP063859.1), pRGF99-1-214k (accession no. NZ_CP075553.1), pBSI128_vf_res (accession no. NZ_MT269849.1), and p205880-2FIIK (accession no. NZ_MN824002.1). The circular map of plasmids was generated with BLAST Ring Image Generator (BRIG). Different regions harboring multidrug resistance are observed between plasmids. (b and c) Alignment of virulence (b) and resistance (c) regions in plasmids pCY814036-iucA, pVir_115011, pSCH6109-Vir, and p130411-38618_1 using Easyfig. Yellow and purple represent antibiotic resistance and virulence genes, respectively. Red represents insertion sequences and transposons. Unidentified open reading frames (ORFs) are shown in blue, and gray shading indicates nucleotide identity.

pCY814036-iucA is a 257,343-bp multireplicon (IncFIB_K_/IncFII_K_) plasmid with a G+C content of 51.4%, which comprised 257 predicted coding DNA sequences (CDSs). Comparative genomic analysis showed that it shared high identity and coverage with plasmids pVir_115011 (GenBank accession no. CP089955), pSCH6109-Vir (GenBank accession no. NZ_CP050860), and p130411-38618_1 (GenBank accession no. MK649826); the differences between plasmids were manifested in the region of drug resistance (Fig. 1a). Importantly, an ~35-kb multidrug resistance (MDR) region in pCY814036-iucA was detected, which carried 12 resistance genes, including *bla*_OXA-10_, *tet*(A), *bla*_LAP-2_, *ANT(3″)-IIa*, *aph(3″)-Ib* (*strA*), *aph(6)-Id* (*strB*), *floR*, *cmlA5*, *qnrS1*, *arr-2*, *sul2*, and *dfrA14*, conferring resistance to β-lactams, aminoglycosides, sulfonamide, fluoroquinolones, tigecycline, and rifampicin. A region of resistance with 99% similarity was also found in plasmid pVir_115011 (GenBank accession no. CP089955) of K. pneumoniae strain WCHKP115011 isolated from West China Hospital. Typically, mobile genetic elements (MGEs), including insertion sequences (ISs) and transposons (Tns) surrounding resistance genes, play an important role in the horizontal transmission of AMR. In plasmid pCY814036-iucA (Fig. 1b), we observed that the *sul2*-*strA*-*strB* cluster, *tet*(A), and *floR* were surrounded by complete IS*5075* and IS*Vsa3* sequences. The second segment, IS*26*–*dfrA14*–*ANT(3″)-IIa*–*bla*_OXA-10_–*cmlA*5–*arr*-*2*–ΔTn*As3*–IS*26*, was located downstream of IS*Vsa3*, which was flanked by IS*26*. Moreover, compared with plasmid pCY814036-iucA, plasmids pSCH6109-Vir and p130411-38618_1 lacked a resistance sequence flanked by ΔTn*As1* (a transposon truncated by the insertion of an IS*26* element) and an IS*26* element, which contained the *bla*_LAP-2_ and *qnrS1* genes. In addition to the resistance genes mentioned above, pCY814036-iucA was found to harbor the aerobactin genes *iucABCD-iutA*. The virulence region exhibited 100% identity with 100% coverage to pVir_115011, pSCH6109-Vir, and p130411-38618_1 (Fig. 1c). The complete sequence of pCY814036-iucA was submitted to oriTfinder, which confirmed that this IncFIB_K_/IncFII_K_ plasmid carried essential conjugative modules, including an origin of transfer (*oriT*) region, a relaxase gene, a type IV coupling protein (T4CP) gene, and a gene cluster for the type IV secretion system (T4SS) (see Fig. S1a in the supplemental material).

10.1128/msphere.00477-22.1 FIG S1 Conjugative systems of plasmids pCY814036-iucA (a) and pCY814036-KPC2 (b). The conjugation modules of the plasmids were predicted by oriTfinder. Download FIG S1, PDF file, 0.1 MB. Copyright © 2022 Xia et al. 2022 Xia et al. https://creativecommons.org/licenses/by/4.0/ This content is distributed under the terms of the Creative Commons Attribution 4.0 International license.

### Genomics of *bla*_KPC-2_- and *rmtB-*coharboring plasmid pCY814036-KPC2.

**FIG 2:**
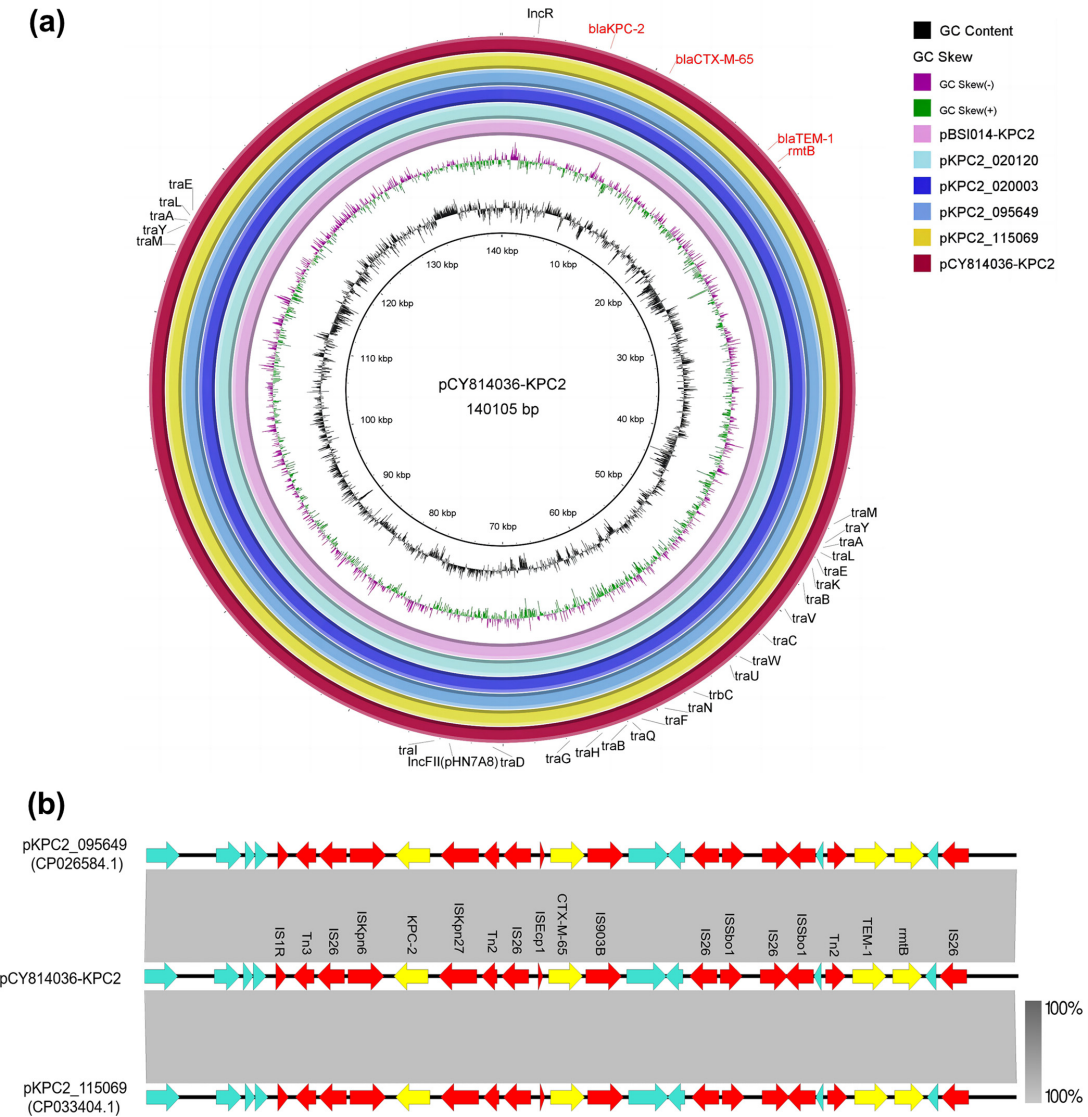
(a) Comparative analysis of six *bla*_KPC-2_-positive plasmids, pCY814036-KPC2 (GenBank accession no. CP093153), pKPC2_115069 (accession no. CP033404.1), pKPC2_095649 (accession no. CP026584.1), pKPC2_020003 (accession no. CP031720.1), pKPC2_020120 (accession no. CP043358.1), and pBSI014-KPC2 (accession no. MT269822.1). The circular map of plasmids was generated with BRIG. (b) Linear comparison of the resistance regions of plasmids pCY814036-KPC2, pKPC2_115069, and pKPC2_095649 using Easyfig. Antibiotic resistance genes are shown in yellow. Genes encoding insertion sequences and transposons are shown in red. Unidentified ORFs are shown in blue, and gray shading indicates nucleotide identity.

The IncFII/IncR-type plasmid pCY814036-KPC2, with a size of 140,105 bp, exhibited a G+C content of 52.8% and comprised 170 predicted coding sequences. A BLASTN search against the NCBI nucleotide database indicated that *bla*_KPC-2_-bearing plasmids of this type are prevalent in K. pneumoniae strains, with 99 to 100% identity and 100% query coverage to each of the following plasmids: pKPC2_115069 (GenBank accession no. CP033404.1), pKPC2_095649 (accession no. CP026584.1), pKPC2_020003 (accession no. CP031720.1), pKPC2_020120 (accession no. CP043358.1), and pBSI014-KPC2 (accession no. MT269822.1) (Fig. 2a). Sequence analysis and annotation revealed that the genetic environment of pCY814036-KPC2 harbored only a resistance region where multiple mobile genetic elements existed, each of which in turn carried antibiotic genes specific for resistance to carbapenem (*bla*_KPC-2_), β-lactam (*bla*_CTX-M-65_ and *bla*_TEM-1_), or aminoglycoside (*rmtB*) antibiotics (Fig. 2b). The four above-described resistance elements of pCY814036-KPC2 were located mainly in three genetic segments. Notably, *bla*_KPC-2_ was harbored by a composite transposon in which IS*Kpn6* and IS*Kpn27* were located downstream and upstream of the *bla*_KPC-2_ gene, respectively. The segment ΔTn*2-*IS*Kpn27*-*bla*_KPC-2_-IS*Kpn6*-IS*26-*ΔTn*3* was linked to the region downstream of IS*1R*. The transfer of the β-lactamase gene *bla*_CTX-M-65_ was mediated by the insertion sequences IS*Ecp1* and IS*903B* through the formation of the cluster IS*Ecp1-bla*_CTX-M-65_-IS*903B* (17). Notably, both IS*Ecp1* and the transposon Tn*2* were truncated by the insertion sequence IS*26*. The third drug resistance segment was flanked by IS*26*, which consisted of the aminoglycoside resistance gene *rmtB* and a truncated Tn*2* transposon, where Tn*2* also carried the *bla*_TEM-1_ gene. Furthermore, oriTfinder analysis showed that plasmid pCY814036-KPC2 carried genes encoding an incomplete conjugative system with a missing T4CP gene (Fig. S1b), which transfers single-stranded DNA (ssDNA) from donor to recipient cells (18).

### Transferability of plasmids carried by K. pneumoniae CY814036 to recipients.

**FIG 3:**
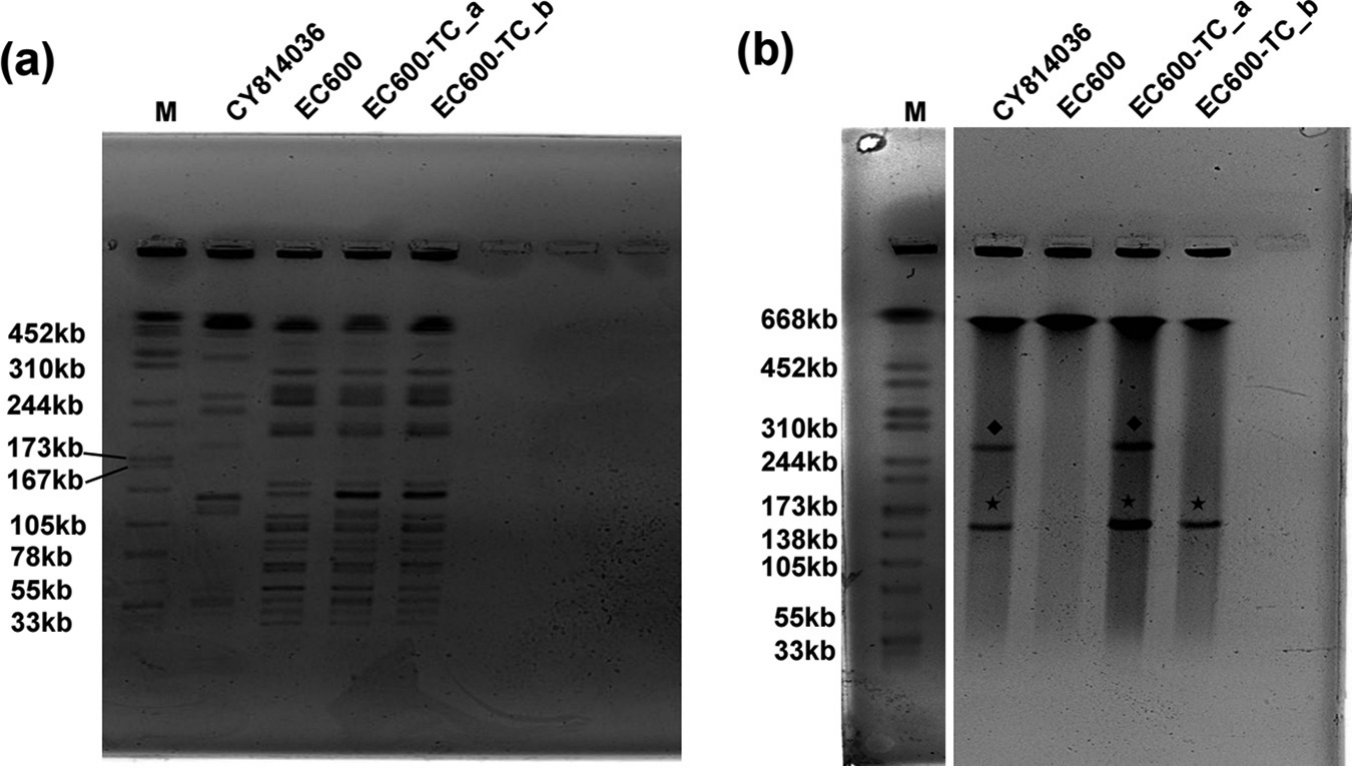
XbaI PFGE (a) and S1-PFGE (b) of K. pneumoniae CY814036 and the corresponding transconjugants. ◆, pCY814036-iucA; ★, pCY814036-KPC2. The same symbol is used to represent the parental strain and its transconjugants. M, molecular weight marker.

Conjugative plasmids make a significant contribution to the fusion of high pathogenicity and carbapenem resistance. To investigate how strain CY814036 acquired drug resistance genes and virulence genes, a conjugation experiment was first conducted to test the potential of plasmids to be transferred to rifampicin-resistant Escherichia coli strain EC600. We observed that the plasmids of CY814036 could be effectively transferred to EC600 (8.0 × 10^−6^), and two transconjugant strains, E. coli EC600-TC_a and EC600-TC_b, were then identified by mass spectrometry (MS) and PCR. An assay targeting the marker genes *bla*_KPC-2_ and *iucA* showed that EC600-TC_a acquired *bla*_KPC-2_ and *iucA*, while EC600-TC_b acquired only *bla*_KPC-2_. We further confirmed that the transconjugants have PFGE profiles similar to that of EC600 (Fig. 3a). Moreover, the results of S1-PFGE showed that strain CY814036 harbored two circular plasmids, designated pCY814036-iucA and pCY814036-KPC2, respectively. Notably, we observed that pCY814036-KPC2 could be transferred to EC600 individually (EC600-TC_b) or together with pCY814036-iucA (EC600-TC_a) (Fig. 3b). Combining the results for the genetic characteristics of the plasmids, we determined that pCY814036-iucA was a self-transmissible plasmid containing antibiotic resistance as well as hypervirulence features, although we did not observe the separate transfer of either resistance or hypervirulence genes.

**FIG 4:**
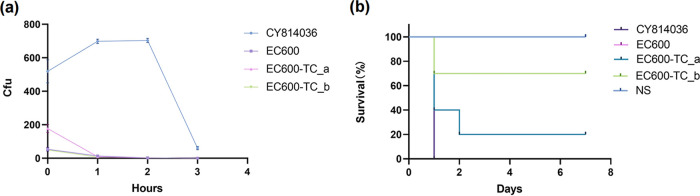
Differential virulence analysis of K. pneumoniae CY814036, EC600, EC600-TC_a, and EC600-TC_b. (a) Serum resistance of the strains. Each test was repeated three times. Data are presented as the means ± SD. No significant difference (*P* > 0.05) was observed among EC600, EC600-TC_a, and EC600-TC_b. (b) Mouse infection model with an inoculum of 1.0 × 10^7^ CFU to depict the differential virulence of strains at 7 days. The survival curves of each group are significantly different (*P* < 0.0001). NS, normal saline.

E. coli EC600-TC_a exhibited an antimicrobial drug susceptibility profile similar to that of CY814036, with resistance to most antibiotics. Although the other transconjugant, EC600-TC_b, carried only the drug resistance plasmid pCY814036-KPC2, it also exhibited high-level resistance to all of the β-lactams antibiotics as well as aminoglycoside antibiotics (Table 1). In addition to the drug resistance phenotype, we evaluated whether the virulence phenotype was transferred to recipient cells. The serum resistance of the transconjugants EC600-TC_a and EC600-TC_b was found to be similar to those of their parental strains (Fig. 4a). However, the acquisition of the pCY814036-iucA plasmid significantly reduced the survival rate of mice. The survival rate of BALB/c mice was only 20% after 7 days of infection with an inoculum of 1 × 10^7^ CFU of strain EC600-TC_a (Fig. 4b).

## DISCUSSION

In recent years, hypervirulent K. pneumoniae (hvKP) has been emerging as a clinically significant pathogen responsible for severe invasive infections, causing high rates of mortality and morbidity in critically ill patients. A previous epidemiological analysis showed that hvKP strains belong mainly to ST23 for the K1 capsular serotype, followed by ST65, ST86, ST375, and ST25 for K2 (19). In this study, we observed an ST25/K2 clinical strain, CY814036, that exhibited a hypermucoviscosity phenotype and coharbored multiple virulence biomarkers, including *iucA*, *iroB*, and *peg-344* (16). It was previously reported that both *rmpA* and *rmpA2* are commonly identified to be located on plasmids of K. pneumoniae, and they play an important role in the resulting hypermucoviscosity phenotype and increasing virulence (20–23), whereas only chromosomal *rmpA* (c-*rmpA*), rather than plasmid-borne *rmpA* and *rmpA2*, contributes to the acquisition of the hypermucoviscosity phenotype in CY814036. The K. pneumoniae strain also harbored *bla*_KPC-2_ resistance elements, thus being characterized as CR-hvKP. To our knowledge, K. pneumoniae strains with ST25, a member of the clonal complex 65 (CC65) family, are widely distributed worldwide, including in Asia, South America, and Africa (24–26). There have also been reports of nosocomial epidemics in China (27). Obviously, this emphasizes the need for tracking the prevalence of the ST25 clone of carbapenemase-producing hvKP in mainland China.

Notably, we confirmed the presence of a conjugative MDR-virulence plasmid, pCY814036-iucA, which increased the risk of the simultaneous transfer of both virulence and AMR determinants in a single event. Nonconjugative pK2044-like plasmids are usually recognized as the most prevalent virulence plasmids of K. pneumoniae (15). In contrast to nonconjugative virulence plasmids, the pCY814036-iucA plasmid is a self-transmissible plasmid that possesses four conjugative modules and could be conjugated to E. coli EC600. In a previous study (13), the first conjugative virulence plasmid, p15WZ-82 Vir, was identified in Klebsiella variicola, and its capacity for self-transduction was shown. Additionally, the virulence plasmid pK2606 in Klebsiella pneumoniae was similarly described by Tian et al., which comprised a complete gene cluster for the type IV secretion system along with the *iucABCD-iutA* genes encoding aerobactin (28). To date, the emergence of conjugative virulence plasmids also encoding antimicrobial resistance or MDR remains uncommon. Zhou et al. described the conjugative plasmid p1864-1 of the IncFIB/IncH1B type, which harbored both resistance genes and the *iuc* operon (29). Many studies have suggested a close relationship between the IncF subtype IncFIB/IncFII plasmids and AMR genes (30–33), whereas a recent study showed that the IncFIB_K_ replicon has a significant impact on promoting the development and replication of virulence plasmids, especially the plasmid-borne *iuc* genes normally harbored by an IncFIB_K_ replicon (34). Therefore, we determined that the mechanism of pCY814036-iucA-like plasmid formation was the acquisition of *iucABCD-iutA* by the IncFIB_K_/IncFII_K_ conjugative plasmid. A striking finding is that similar virulence plasmids have been transferred between K. pneumoniae strains of different STs (34). Furthermore, a linear alignment showed that the variations between pCY814036-iucA and other similar plasmids were situated in the MDR region, indicating that drug resistance in these plasmids is continuously evolving via transposition or recombination. Data from previous studies are still insufficient to characterize the MDR region harbored by pCY814036-iucA. We determined that the active transmission of resistance genes prompted the evolution of pCY814036-iucA into a conjugative MDR-hypervirulence plasmid. Notably, target site duplications (TSDs) flanking IS*26* were not recognized in this region, implying the acquisition of drug resistance genes via homologous recombination. Recombination events mediated by insertion sequences might be responsible for the convergence of plasmids carrying MDR and virulence factors, which raises huge concerns about the occurrence and spread of these plasmids and the effectiveness of current protocols for controlling such K. pneumoniae strains.

We also demonstrated that K. pneumoniae CY814036 carries an IncFII/IncR-type plasmid, pCY814036-KPC2, with several antibiotic resistance determinants, including β-lactams resistance genes (*bla*_KPC-2_, *bla*_CTX-M-65_, and *bla*_TEM-1_) and an aminoglycoside resistance gene (*rmtB*). To date, the acquired *bla*_KPC-2_ gene is the gene most widely reported among CRKP isolates involved in hospital outbreaks, which is commonly harbored by IncFII/IncR-type plasmids and located on non-Tn*4401* elements (NTE_KPC_), as reported in China (15). In this study, the *bla*_KPC-2_-bearing transposable unit was a movable element belonging to the transposon Tn*6296* and its derivatives, which play a very important role in transferring the *bla*_KPC-2_ gene. Notably, Tn*2* and Tn*3* were truncated by two IS*26* insertion sequences in the Tn*6296-*like transposon harbored by pCY814036-KPC2, which was different from NTE_KPC_. The β-lactamase gene *bla*_CTX-M_ is usually transferred by IS*Ecp1* and IS*CR1* (17); we noticed that the *bla*_CTX-M-9_ group gene *bla*_CTX-M-65_ was transferred by IS*Ecp1* and IS*903B* together (35). *bla*_CTX-M-65_ is continuously prevalent in Escherichia coli, which may be due to the spread of plasmids carrying *rmtB* (36). The *rmtB* gene mainly encodes a 16S rRNA methyltransferase to mediate a high level of resistance to aminoglycoside antibiotics (37). We observed that the *rmtB* gene is commonly located downstream of the transposon Tn*2* to form the Tn*2*-*rmtB* component, which is related to the *bla*_TEM-1_ gene and positioned upstream of *rmtB* (38). Our analysis described above shows that *bla*_KPC-2_, *bla*_CTX-M-65_, *bla*_TEM-1_, and *rmtB* were all carried by typical mobile genetic elements, which potentially contributed to the dissemination of resistance genes. Interestingly, pCY814036-KPC2 harbored an incomplete conjugative system, whereas it was transferred from CY814036 to EC600 alone or together with pCY814036-iucA. This phenomenon indicated that pCY814036-KPC2 has the potential to be mobilized, which could be realized with the help of the conjugative plasmid pCY814036-iucA (18).

Importantly, our study confirmed the efficient transfer of two plasmids, pCY814036-iucA and pCY814036-KPC2, which thus conferred multidrug resistance to E. coli EC600. Russo et al. demonstrated previously that the aerobactin gene *iucA* is a significant virulence-associated determinant of hvKP (16). We evaluated whether the acquisition of plasmid pCY814036-iucA could confer a hypervirulence phenotype. Nevertheless, the survival rate of mice indicated that obtaining the conjugative virulence plasmid may not produce virulence as high as that of CY814036, but in comparison to E. coli EC600 and EC600-TC_b, transconjugant EC600-TC_a was endowed with high-level virulence in mice. Therefore, the discovery that the pCY814036-iucA-like plasmid not only can effectively self-transfer among clinical organisms but also confers the MDR-virulence phenotype needs to raise concern for public health and effective action to prevent hospital outbreaks.

In conclusion, we investigated the microbiological features and genetic background of an ST25 clinical strain (CY814036) of K. pneumoniae with resistance to carbapenem and a hypermucoviscosity phenotype. Strain CY814036 coharbored two conjugative plasmids: one is a novel hybrid plasmid bearing virulence genes and an ~35-kb MDR region, and the other carries both *bla*_KPC-2_ and *rmtB*. Notably, various MGEs surrounding resistance elements can mediate active genetic recombination or transposition events, which significantly increases the likelihood of the dissemination of these resistance phenotypes. Moreover, the cotransfer of two plasmids harbored by strain CY814036 could promote the convergence of Klebsiella pneumoniae clones that are both carbapenem resistant and hypervirulent, the evolutionary trend of which is important to constantly monitor in China.

## MATERIALS AND METHODS

### Bacterial strain.

The Klebsiella pneumoniae strain (designated CY814036) characterized in our research was identified at a tertiary teaching hospital in Chongqing, China, in 2018. The strain identity was established using a matrix-assisted laser desorption ionization–time of flight (MALDI-TOF) mass spectrometry apparatus (Bruker, Germany), and the strain was stored in LB broth containing 50% glycerol at −80°C. Subsequently, using previously described primers (16), the virulence genes *iucA*, *iroB*, p-*rmpA*, p-*rmpA2*, and *peg-344* as well as the resistance gene *bla*_KPC-2_ were detected by PCR analysis of this strain.

### Antimicrobial susceptibility tests.

The MICs of the strains were determined for 11 antimicrobial agents using the broth microdilution method and interpreted in terms of the breakpoints recommended by CLSI document M100 (39). The antimicrobial agents tested included amikacin, aztreonam, ceftriaxone, ceftazidime, cefepime, ciprofloxacin, ceftazidime-avibactam, levofloxacin, imipenem, meropenem, and tigecycline. U.S. Food and Drug Administration (FDA) (40) standards were employed to determine the breakpoint of tigecycline against *Enterobacteriaceae*. E. coli strain ATCC 25922 was used as a quality control strain.

### Genome sequencing and bioinformatics.

Whole-genome sequencing (WGS) was performed on Klebsiella pneumoniae strain CY814036, and the genomic DNA was extracted using the Wizard genomic DNA purification kit (Promega). The extracted DNA was used to construct one PacBio SMRT bell library, and 10-kb long-read sequencing was performed by using the PacBio RS II SMRT platform. The genomic DNA was also subjected to library preparation by using a NEXTflex rapid DNA sequencing (DNA-Seq) kit for Illumina and then sequenced using the 150-bp paired-end Illumina (San Diego, CA) HiSeq platform. The CY814036 genome was sequenced with over 100-fold coverage using both platforms. Notably, the Illumina data were used to evaluate the complexity of the genome. Raw reads were trimmed, filtered, and then assembled into a contig using Unicycler v0.4.8 (41). Finally, error correction of the assembled genome sequences was performed using Pilon v1.22 software (42). Gene prediction was conducted by using Glimmer (43), GeneMarkS software (44), and BLAST.

The PlasmidFinder database was used to identify plasmid replicons with a minimum identity of 95% and a minimum coverage of 60% (https://cge.food.dtu.dk/services/PlasmidFinder/). Multilocus sequence typing (MLST) and capsular typing were performed using MLST version 2.0 software (https://cge.food.dtu.dk/services/MLST) and Kaptive, respectively. The acquired antibiotic resistance genes were located by employing CARD (https://card.mcmaster.ca/analyze/rgi). Putative virulence factors were identified using the Virulence Factors Database (VFDB) (available at http://www.mgc.ac.cn/VFs/main.htm). The insertion sequences (ISs) and transposons (Tns) were identified using ISFinder software (https://www-is.biotoul.fr/blast.php). In addition, the conjugation module of plasmids was predicted by oriTfinder (https://tool-mml.sjtu.edu.cn/oriTfinder/oriTfinder.html). Sequence similarity comparison of plasmids was performed using BLAST, and the alignment of plasmids with highly homologous structures was then visualized by using BLAST Ring Image Generator (BRIG) version 0.95 and Easyfig v2.2.5.

### Conjugative plasmid transfer.

Experiments involving conjugative plasmid transfer were carried out using K. pneumoniae strain CY814036 as the donor and E. coli EC600 (rifampicin resistant) as the recipient. Both the donor isolate and the recipient strain were grown to logarithmic phase (optical density [OD] of approximately 0.6) at 37°C at 220 rpm in 5 mL of fresh LB broth. In the next step, 300-μL cultures of the donor cells and 600-μL cultures of the recipient cells were mixed and inoculated into 4 mL LB broth at 37°C overnight. The 100-μL mixture was inoculated onto Mueller-Hinton (MH) agar plates containing 0.5 μg/mL meropenem and 600 μg/mL rifampicin. After incubation at 37°C overnight, transconjugants that grew on the selective medium were collected and identified using mass spectrometry (MS) and PCR for the presence of vital genes. XbaI pulsed-field gel electrophoresis (PFGE) and S1 nuclease PFGE (S1-PFGE) were performed to confirm the transfer of plasmids, as previously described (45, 46). The number of transconjugants was divided by the total number of recipient cells to determine the conjugation efficiency.

### Serum resistance assay.

Serum was obtained from 5 healthy individuals and stored at −80°C. Briefly, mid-log-phase bacterial cells were diluted in LB broth to 1 × 10^6^ CFU/mL and mixed with serum at a 1:3 proportion. Each mixture was then incubated at 37°C at 200 rpm for 3 h (checked at 0, 1, 2, and 3 h). The test was performed at least three times independently, and the percentage of CFU counts was characterized as the result of serum resistance, which was graded from 1 to 6. Unpaired two-sided Student’s *t* test was performed for strains. Data are presented as the means ± standard deviations (SD). Using normal human serum, a strain is generally considered to be resistant if it can reach grades 5 to 6, as previously described (47).

### Mouse infection model.

The virulence potential of strains was evaluated using a mouse infection model as described following. Five-week-old male BALB/c mice weighing an average of 16 g were obtained from Hunan SJA Laboratory Animal Co., Ltd. (Hunan, China), and were given access to food and water during the experiment. Ten mice in each group were inoculated intravenously with 100 μL of bacteria at a concentration of 1 × 10^7^ CFU/mL. It was decided that the normal saline (NS) group would serve as the control group. For up to 7 days, death rates and symptoms were monitored. The virulence of strains was determined by the survival of the inoculated mice. Survival curves were generated by using Prism 9. Statistical analysis was performed using the log rank (Mantel-Cox) test. Animal ethics approval was obtained from the Ethics Committee of the First Affiliated Hospital of Chongqing Medical University.

### Data availability.

The genomic data for the chromosome of K. pneumoniae CY814036 and plasmids pCY814036-iucA and pCY814036-KPC2 were submitted to GenBank under accession no. CP093151, CP093152, and CP093153, respectively.
