# Supplementary material for: Coexistence of Multidrug Resistance and Virulence in a Single Conjugative Plasmid from a Hypervirulent Klebsiella pneumoniae Isolate of Sequence Type 25
Source: mSphere. 2022 Dec 6;7(6):e00477-22. doi: 10.1128/msphere.00477-22 (PMC9769751; doi:10.1128/msphere.00477-22)
Supplement: TABLE S1 [file msphere.00477-22-s0001.pdf]

Virulence factors	Related genes	Start…Stop(bp)
Type 3 fimbriae	*mrkA*	855621...856229
	mrkB	856325...857026
	mrkC	857038...859524
	mrkD	859515...860510
	mrkF	860524...861159
	mrkH	862644...863348
	mrkI	862054...862626
	mrkJ	861194...861910
Type I fimbriae	*fimA*	848248…848796
	fimB	850350…850955
	fimC	846887…847591
	fimD	724238…726832; 844193…846805
	fimE	849276…849884
	fimF	843655…844182
	fimG	843142…843642
	fimH	842219…843127
	fimI	847641…848177
	fimK	840810…842222
Capsule	-	1670181…1671077; 1671470…1672099; 1673061…1674494; 1674640…1675776; 1676231…1678399; 1680692…1681858; 1681815…1683104; 1683122…1684579; 1687120…1688517; 1688681…1690087; 1690330…1691745; 1691768…1693138; 1693302…1694468; 5234159…5235223
LPS rfb locus	-	1697462…1698229; 1698229…1698969; 1698985…1700883; 1700896…1702050; 1702047…1702940; 1702953…1704083; 1704179…1704179
PEG344	*peg-344*	1806315…1806823
RcsAB	*rcsA*	1865936…1866559
	rcsB	1520495…1521145
RmpA	*rmpA*	1804603…1805235

Virulence factors	Related genes	Start…Stop(bp)
Ent siderophore	*entA*	3874523…3875278
	entB	3875278…3876129
	entC	3877760…3878935
	entD	3892431…3893054
	entE	3876143…3877750
	entF	3884545…3888426
	entS	3880339…3881580
	fepA	3890136…3892364
	fepB	3879124…3880083
	fepC	3883686…3884480
	fepD	3881693…3882700
	fepG	3882697…3883689
	fes	3888668…3889876
Salmochelin	*iroB*	1813271…1814386
	iroC	1809489…1813133
	iroD	1808154…1809383
	iroE	2751460…2752389
	iroN	1815258…1817432; 3148045…3150228
Yersiniabactin	*fyuA*	1824738…1826759
	irp1	1830368…1839859
	irp2	1839947…1846054
	ybtA	1846245…1847204
	ybtE	1826890…1828467
	ybtP	1847461…1849173
	ybtQ	1849160…1850962
	ybtS	1852263…1853567
	ybtT	1828471…1829274
	ybtU	1829271…1830371
	ybtX	1850955…1852235
Aerobactin	*iutA*	3293739…3295928
AcrAB	*acrA*	4130534…4131727
	acrB	4131750…4134896

Virulence factors	Related genes	Start…Stop(bp)
T6SS-I	-	3052527…3053306
	clpV/tssH	3055677…3058331
	dotU/tssL	3060795…3061484
	hcp/tssD	3058596…3059087
	ompA	3059092…3060798
	sciN/tssJ	3039571…3040113
	tli1	3047147…3047677; 3047746…3048276; 3048344…3048871; 3048939…3049469; 3049538…3050068; 3050137…3050667; 3050736…3051266; 3051335…3051865; 3051933…3052463
	tssF	3041140…3042894
	tssG	3040091…3041176
	vasE/tssK	3061481…3062824
	vgrG/tssI	3053306…3055684
	vipA/tssB	3064421…3064912
	vipB/tssC	3062834…3064378
T6SS-II	*clpV*	1208839…1211412
T6SS-III	-	2067397…2069424
	-	2071523…2072734
	-	2081638…2082552
	-	2082636…2082965
	dotU	2061951…2062604
	icmF	2072749…2076165
	impA	2080091…2081506
	impF	2079628…2080071
	impG	2076299…2078062
	impH	2078062…2079108
	impJ	2060614…2061954
	ompA	2062608…2064305
	sciN	2079089…2079625
	vgrG	2064767…2067394
