# Supplementary figures and images for: Coexistence of Multidrug Resistance and Virulence in a Single Conjugative Plasmid from a Hypervirulent Klebsiella pneumoniae Isolate of Sequence Type 25
Source: mSphere. 2022 Dec 6;7(6):e00477-22. doi: 10.1128/msphere.00477-22 (PMC9769751; doi:10.1128/msphere.00477-22)

(a)

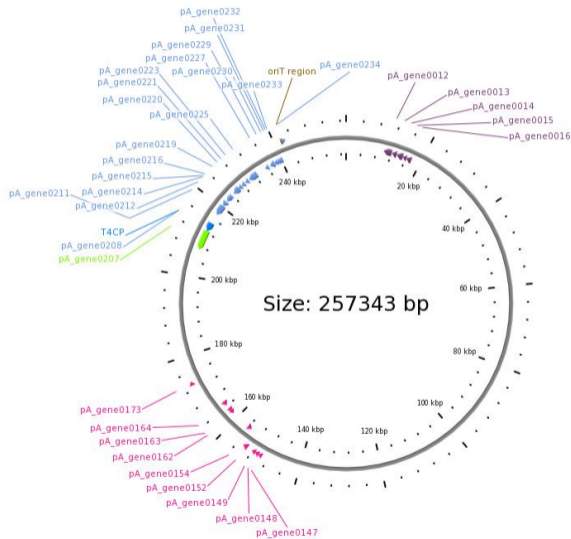

(b)

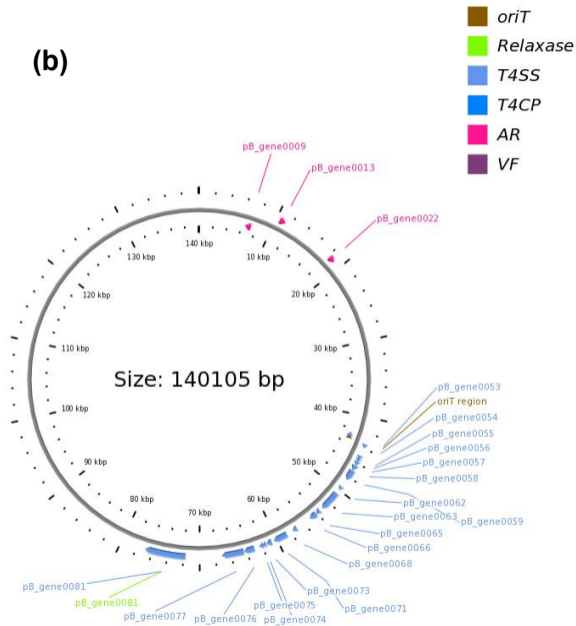

Supplement: FIG S1 [file msphere.00477-22-s0002.pdf]
